# Factors associated with sleep quality during chemotherapy: An integrative review

**DOI:** 10.1002/nop2.516

**Published:** 2020-06-09

**Authors:** Regina Claudia da Silva Souza, Maiara Rodrigues dos Santos, Izabel Alves das Chagas Valota, Cristina Silva Sousa, Ana Lucia Siqueira Costa Calache

**Affiliations:** ^1^ University of São Paulo School of Nursing Sao Paulo Brazil; ^2^ Sirio Libanes Hospital Sao Paulo Brazil; ^3^ Department of Medical‐Surgical Nursing University of São Paulo School of Nursing Sao Paulo Brazil

**Keywords:** cancer, chemotherapy, nurses, nursing, predisposing factors, sleep quality

## Abstract

**Aim:**

To identify the most important factors associated with sleep pattern changes in patients with cancer during chemotherapy treatment.

**Design:**

An integrative review of the literature was performed between December 2017–August 2018.

**Methods:**

Two independent reviewers searching the National Library of Medicine (PubMed/MEDLINE), Cumulative Index of Nursing and Allied Health Literature (CINAHL) and Latin American and Caribbean Literature in Health Sciences (LILACS), Scopus and Scielo. The process followed the recommendations of the PRISMA tool. A total of 16 articles were selected for the final study sample, including 11 cohort studies and 5 cross‐sectional studies.

**Results:**

The predisposing factors for the most prevalent sleep disturbances were precipitants related to the disease and the treatment, such as fatigue, pain, depression, anxiety and distress. Predisposing factors related to lifestyle and demographic characteristics have a significant correlation with sleep disturbances.

## 1 INTRODUCTION

The number of cancer cases has been growing worldwide, and cancer is among the leading cause of death in most countries. Eighteen million new cases of cancer occurred in the world in 2018 and 9.8 million deaths and that cancer will become the leading cause of mortality in all countries (Brasil, 2019).

As cancer is a relevant global public health problem, its epidemiological profile has been gaining attention. In Brazil, the National Cancer Care Policy was established by Decree no. 2439/GM of 8 December 2005; the policy involves convergent lines of action that emphasize health promotion, disease prevention, diagnosis, treatment, rehabilitation, palliative care, quality of life and research (Brasil, 2019; Sawada et al., 2016).

In cancer treatment, surgical, radiotherapeutic, chemotherapeutic or biological approaches through hormones, antibodies or growth factors that act locally or systemically are employed. Chemotherapeutic agents are the most common treatment mode among all patients. Such agents frequently cause multiple symptoms that occur simultaneously and are associated with a reduction in quality of life. Common symptoms include pain, nausea, vomiting, fatigue, depression and sleep pattern changes symptoms have the high prevalence (Miaskowski, 2014; Palesh et al, 2018; Ratcliff, Lam, Arun, Valero, & Cohen, 2014).

Sleep disorders are common in cancer patients undergoing chemotherapy and coexist with many other disabling symptoms (Induru & Walsh, 2014). Periods of sleep deprivation cause a decrease in immune function, increased levels of pro‐inflammatory factors, a reduction in glucagon levels, dysregulation of emotional and cognitive skills, a significant drop in quality of life scores and aggravation of pre‐existing health problems (Besedovsky, Lange, & Born, 2012).

Studies show that 30%–60% of oncology patients present with a sleep disorder, with insomnia being the most common (Palesh et al., 2012). The factors that influence these disorders can be classified as predisposing, precipitating and perpetuating, according to the 3Ps’ model (Spielman, Caruso, & Glovinsky, 1987). Predisposing factors are enduring traits, such as female sex, advanced age or psychiatric history, that are known to increase the individual's susceptibility to insomnia. Precipitating factors include stressful events that occur during treatment, such as diagnosis, disease recurrence, effects of disease and treatment and late effects or other symptoms. Perpetuating factors are those that maintain sleep disturbances after the resolution or adaptation of precipitating factors, such as maladaptive behaviours and sleep‐related thoughts (Miaskowski et al., 2014).

The Oncology Nursing Society identified sleep pattern change as a research priority (Knobf et al., 2015). Although chemotherapy is a common treatment for oncology patients, research on disrupted sleep during this treatment is limited. A 2010 meta‐synthesis of studies on this subject in women demonstrated this knowledge gap; the included studies had relatively small sample sizes, were limited to a specific type of tumour (breast cancer), did not assess predictors of the sleep disorder and assessed the problem at a limited number of time intervals (Enderlin et al., 2010; Mark et al., 2017). These limitations affected the quality of the evidence, led to inconsistent results and are indicators of the need for new studies with greater methodological rigour.

An understanding of the relationship between chemotherapy treatment and sleep pattern changes is essential because it will inform the development of interventions that health professionals can implement to control the factors that affect sleep quality in this population and will support evidence‐based practice. Understanding these aspects is essential for defining interventions that can improve quality of life during chemotherapy and prevent further damage and increased severity in patients. With this perspective, an integrative review was conducted to identify the most important factors associated with sleep pattern changes in patients with cancer during chemotherapy treatment. The guiding question for achieving this objective was “What are the factors associated with sleep pattern changes in cancer patients during chemotherapy?”.

## 2 AIM AND METHODS

An integrative review of the existing scientific literature about the factors associated with sleep pattern changes in cancer patients during chemotherapy was conducted. The procedure followed the recommendations of the PRISMA tool (Moher, Liberati, Tetzlaff, & Altman, 2009). This research method allows the pre‐existing literature on a given topic or question to be summarized in a systematic and orderly manner, which will support professionals in decision making, assist in improving clinical practice and reveal gaps in knowledge production that need to be filled with new studies (Souza, Silva, & Carvalho, 2010).

The articles were analysed in six stages: selection of the research question; selection of studies according to inclusion and exclusion criteria; presentation of the characteristics and categorization of the studies; analysis of the studies; interpretation of the results; and presentation of the knowledge synthesis (Souza et al., 2010).

The review was conducted between December 2017–August 2018 by two independent reviewers. The search of the databases was performed by one of the researchers and was based on the descriptors defined according to the research question. After this step, two reviewers independently analysed the articles in all stages. Disagreements between the reviewers about the selected articles were resolved by a third reviewer.

### 2.1 Search strategy

The following databases were accessed: National Library of Medicine (PubMed/MEDLINE); Cumulative Index of Nursing and Allied Health Literature (CINAHL); and Latin American and Caribbean Literature in Health Sciences (LILACS), Scopus and Scielo.

DeCS Health Science Descriptors used were in three languages (Portuguese: “quimioterapia”, “agentes antineoplásicos”, “sono”, “transtornos do sono” and “neoplasia”; English: “chemotherapy”, “neoplastic agents”, “sleep disorders”, “sleep disturbance”, “sleep”, “neoplasms”; and Spanish: “transtornos del sueño”, “sueño”, “antineoplasicos” and “neoplasias”). The keywords sleep, chemotherapy and neoplasia were also used in the three languages in databases where the combination of the descriptors did not return any results. The search queries used a combination of two or three descriptors or keywords in each database with the Boolean operator AND (Table S1).

### 2.2 Eligibility criteria

The inclusion criteria established for article selection were full texts available in the selected databases without limits about the year of publication and published in English, Portuguese or Spanish; primary studies with quantitative designs including cancer patients undergoing chemotherapy; studies that used objective and subjective measurements for sleep evaluation; and studies that included an analysis of factors related to changes in the sleep pattern. The first step, which consisted of querying the databases according to the elaborated strategies, occurred between December 2017–January 2018. We adopted the definition of sleep disorders of the American Academy of Sleep Medicine, which includes four categories: dyssomnias, which include disorders of initiating sleep; parasomnias, which include manifestations of central nervous system activation; disorders associated with mental, neurological or medical disorders; and sleep disorders that include those without a defined aetiology (American Academy of Sleep Medicine, 2001).

Studies were excluded if they were conducted with relatives and/or caregivers; conducted on laboratory animals; focused on paediatric patients; had as the primary outcome fatigue, distress, depression, mood and quality of life; and did not evaluate sleep using objective and/or subjective instruments. The Joanna Briggs Institute framework was used to classify the level of evidence of the studies (Joanna Briggs Institute, 2013).

### 2.3 Data extraction

**FIGURE 1:**
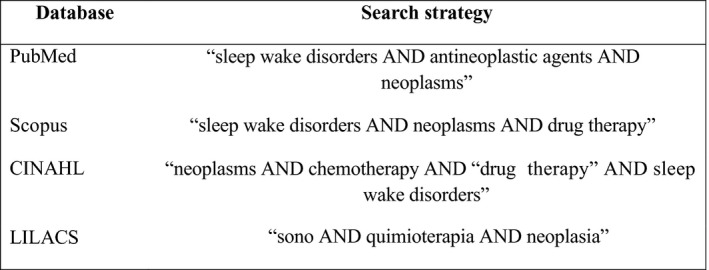
Database/portal and search strategy

The publications were examined first by reading the title and abstract, the studies that met the inclusion criteria were selected for full reading. The texts selected in full had their data related in an instrument built by the researchers with the following variables: identification of the article, objective of the study, name of the authors, year of publication, training of the authors, study design, population/sample, location of the study, sleep assessment instrument used, level of evidence, results (risk factors) and conclusions. Allowing a synthesis and easier analysis of each study (Figure 1).

## 3 RESULTS

**FIGURE 2:**
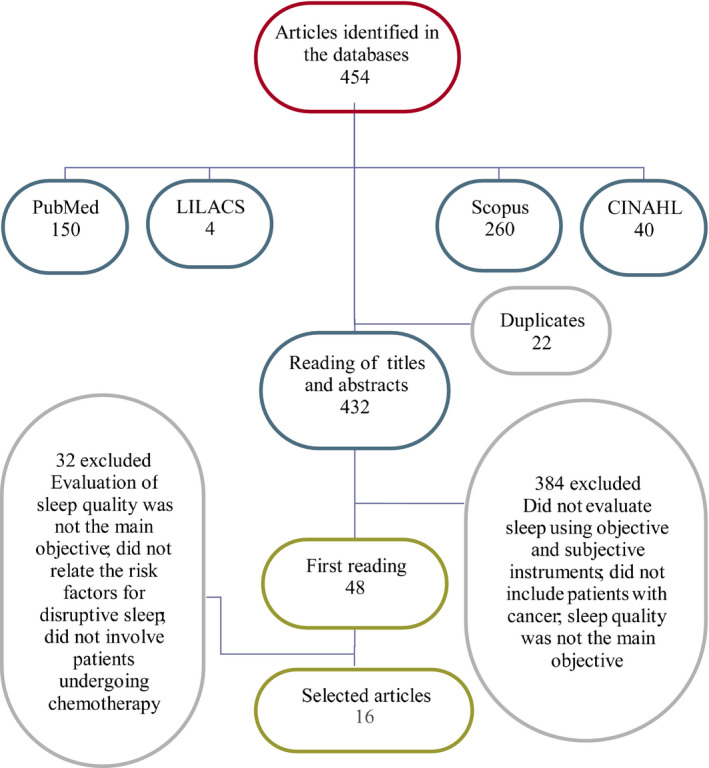
Flow diagram

The search resulted in 454 articles (Figure 2). Among the 454 articles found, those that did not meet the inclusion criteria were excluded, which resulted in 48 articles. Of these, all had the full text available in Portuguese, English or Spanish. About duplication, 22 articles were repeated in the selected databases; thus, 432 were subjected to abstract analysis. A total of 16 articles were selected for the final sample. Among these articles, nine (56.2%) were cohort studies, five (31.2%) were cross‐sectional studies and two (12.5%) were cohort studies with a control group.

About the subjective and objective sleep evaluation instruments used to obtain the data, 10 studies (62.5%) used the Pittsburgh Sleep Quality Index (PSQI), eight (50.0%) used actigraphy and 2 (10.0%) used a sleep diary and the General Sleep Disturbance Scale. The Athens Insomnia Severity Index, Bergen Insomnia Scale, Epworth Sleepiness Scale, Insomnia Severity Index and WHI Sleep Disturbance Scale were among the instruments used in the studies. Of these, six articles (37.5%) combined the use of the PSQI and actigraphy. Three studies (15%) used only actigraphy. The mean number of instruments per study was 1.5, and five studies (31.2%) used objective and subjective measures in combination.

The countries with the most publications on the subject were the United States with nine studies (56.2%), followed by China with two studies (12.5%), Italy with two studies (12.5%) and Brazil, Australia and Japan with one study each (18.7%) (Table 1).

**TABLE 1:** Characteristics of the selected articles and evidence hierarchy

SI	Study	Authors' profession	Study design	Population/sample	Associated factors	Study site	Evidence classification
E2	Rissling et al. (2011)	Doctors	Cohort	69 women	Linear mixed model that showed that depressive symptoms of menopause are associated with changes in sleep patterns (*r* = −.32 and *p* = .035).	USA	3e
E6	Wu, Davis, Padiyar, and Yarandi (2011)	Nurses	Cohort with control group	62 women	Patients with cancer‐related fatigue reported a significantly higher sleep latency (*p* = .041) and poorer sleep quality (*p* = .005) when compared to healthy patients by the Wilcoxon test.	USA	3c
E8	Bischel et al. (2016)	Nurses and doctors	Cohort	1,343 patients	In the regression analysis, the factors associated with poor sleep quality were White race, lower functional status, and higher number of comorbidities. For young patients, these factors were added to living alone, having child care responsibilities and breast cancer.	USA	3e
E9	Nishiura et al. (2015)	Doctors	Cross‐sectional	50 patients	Correlations between sleep disorders and the predictive factors distress, fatigue, worse quality of life and pain were significant (*p* = .01).	Japan	4b
E11	Phillips et al. (2012)	Doctors	Cohort	330 patients	Regression analysis showed that low educational level (*p* = .036), higher number of comorbidities (0.017), previous radiotherapy (0.017), smoking and reduced physical activity (*p* = .015) are associated with poorer sleep quality.	USA	3e
E16	Mansano‐Schlosser et al. (2012)	Nurses	Cross‐sectional	140 patients	Logistic regression analysis showed an association between pain (*p* < .001) and pain intensity (*p* < .001) with poor sleep quality.	Brazil	4b
E20	Jim et al. (2011)	Doctors	Cohort	80 women	Mixed model analysis in which the most recent surgery time was significantly related to daytime activity (*r* = .49, *p* < .01) and to daytime sleep (*r* = −.31, *p* < .01) after chemotherapy. Cancer recurrence was associated with less time in bed at night (*r* =−.26, *p* < .05).	USA	3e
E22	George et al. (2015)	Not informed	Cross sectional	56 patients	Insomnia was correlated with depression score (Pearson's correlation, *r* = .39, *p* = .003) and pain intensity (*r* = .37, *p* = .006). In the regression analysis, only pain was predictive of insomnia (*p* = .047)	Australia	4b
E23	Jim et al. (2013)	Doctors	Cross sectional	78 women	The study participants who slept poorly reported fatigue peaks according to bivariate regression analysis (*p* < .01).	USA	4b
E25	Romito et al. (2014)	Doctors and psychologists	Cross sectional	403 patients	The logistic regression analysis showed that reduced overall quality of life (*p* = .030), reduced physical functioning (*p* = .04), and psychological distress (*p* = .000) were the most significant factors associated with the presence of sleep disorders	Italy	4b
E34	Saini et al. (2013)	Nurses	Cohort	173 patients	Restless legs syndrome was correlated with poorer sleep quality (*p* = .007).	Italy	3e
E38	Chen et al. (2008).	Nurses and doctors	Cohort	115 patients	Cognitive impairment (*p* = .002) and poor functioning (*p* = .01) were associated with poorer sleep quality.	China	3e
E40	Beck et al. (2010)	Nurses and doctors	Cohort	183 women	More advanced age was associated with longer night awakenings (*p* < .001).	USA	3e
E41	Tian et al. (2015)_	Not informed	Cohort with control group	228 participants	Factors associated with poor sleep quality were high level of distress (*p* = .045), depression (*p* = .028), anxiety (*p* = .027), high level of neurotoxicity (*p* = .016) and concomitant chemotherapy and radiotherapy treatment (*p* = .017).	China	3c
E46	Berger et al. (1999).	Nurses	Cohort	72 women	High levels of fatigue were associated with changes in sleep patterns during chemotherapy cycles (*p*‐values of 0.001 and 0.003, respectively).	USA	3e
E47	Liu et al. (2012)	Doctors	Cohort	97 women	The mixed model showed that fatigue is associated with poorer sleep quality (*p* < .01).	USA	3e

Abbreviation: SI, Study Identification.

The studies included populations undergoing chemotherapy for the following cancer types: breast (25.0%), lung (12.5%), gynaecological (12.5%) and cervical (6.25%). In 37.5% of the studies, the sample was not restricted to a specific cancer type. Among all of the studies, 37.5% were conducted by doctors, 18.7% by nurses, 6.25% by psychologists and 18.75% by doctors and nurses together (Table 1). None of the factors analysed in the studies was classified as a perpetuating risk. The analysed factors were grouped as predisposing or precipitating risks only, as shown in Table 2.

**TABLE 2:** Factors related to sleep disturbances

Factors influencing sleep quality during chemotherapy		
Predisposing factors	Worse physical functioning	White race
	Advanced age	Lower educational level
	Caring for small children	Greater number of comorbidities
	Cancer recurrence	Low physical activity
	Living alone	Smoking
	Worse quality of life	Worse cognitive function
	Previous surgery	Restless legs syndrome
Precipitating factors	Presence of pain	Depressive symptoms
	Distress	Previous radiotherapy
	Radiation therapy combined with chemotherapy	Neurotoxicity
	Fatigue	Pain intensity
	Breast cancer	Recent surgery
	Depression	Anxiety

Among the studies that investigated predisposing factors, a significant association was observed between these factors and changes in sleep pattern and quality. In the study by Bischel et al. (2016), both advanced age and younger age, along with poorer physical functioning, more comorbidities, White race and social responsibilities were associated with a greater occurrence of sleep disturbances. Compromised quality of life was significantly correlated with the presence of sleep disturbances in two studies (Nishiura, Tamura, Nagai, & Matsushima, 2015; Romito et al., 2014) but was confirmed as a risk factor by logistic regression analysis only in the study of Romito et al. (2014). Altered physical and cognitive functioning also showed a significant correlation with altered sleep patterns and were identified along with precipitating factors such as distress, pain, depression, fatigue (Berger et al, 1999) and dyspnoea. Number of comorbidities and White race were significantly associated with changes in sleep patterns in two studies (Bischel et al., 2016; Phillips, Jim, Donovan, Pinder‐Schenck, & Jacobsen, 2012). Other factors, including restless legs syndrome, smoking, low physical activity, previous surgery, low functional scale scores and social conditions (living alone and caring for children) were described in only one study each.

Among the studies that described precipitants of changes in sleep pattern, a significant association with fatigue, depression and pain symptoms was confirmed by logistic regression analysis. In studies that assessed pain, the intensity of the symptoms was significantly correlated with sleep disturbances (George, Elias, & Shafiei, 2015; Jim et al., 2011; Mansano‐Schlosser & Ceolim, 2017). Distress and anxiety symptoms were associated with the presence of sleep disturbances (Tian, Chen, & Zhang, 2015).

## 4 DISCUSSION

The present review showed aspects relevant to sleep disturbances in cancer patients undergoing chemotherapy as for this population, sleep disturbances have a strong impact on quality of life and may persist for years after the end of treatment (Mercadante et al., 2017). A detailed understanding of the factors related to sleep disturbance allows greater possibilities for intervention and provides several perspectives on providing care to improve the quality of the sleep pattern. Changes in sleep patterns in cancer patients have a negative effect on disease prognosis, survival and treatment efficacy (Innominato et al., 2012). Thus, prevention strategies and sleep assessment tools need to be included in clinical care.

All of the selected studies used an observational methodology, which, according to the classification of evidence of Joanna Briggs Institute (2013), represents the intermediate level in the hierarchy of evidence. Of these studies, only two used a control group, which is a factor that adds greater robustness to the results. This is a relevant finding that reinforces the need for research with better levels of evidence and greater methodological rigour to explain the nature of the problem. Although they are not classified as the best evidence, observational studies are increasingly considered in health decision‐making, especially considering that under certain conditions, interventional studies are not always feasible (Fronteira, 2013). The use of research designs with comparison groups, follow‐up over multiple periods of time, a large number of participants, rigorous confidence intervals, etc., are characteristics of rigorous studies that the JBI benchmarks of evidence do not consider. Therefore, the JBI classifications of evidence are contested due to their heterogeneity and non‐inclusion of these dimensions (Glasziou, Vandenbroucke, & Chalmers, 2004). However, a study's methodological quality must be deemed satisfactory according to a series of criteria. This aspect is noted in the expert consensus and presented in the guideline for clinical practice published by the Cancer Journey Advisory Group of the Canadian Partnership Against Cancer (Howell et al., 2013). Due to the weakness of the evidence from the studies available in the literature, this guideline presents recommendations for the prevention, screening, evaluation and treatment of sleep disturbances in adults with cancer.

The factors classified as precipitants were the most commonly identified, especially in combination with one another (Nishiura et al., 2015; Romito et al., 2014; Tian et al., 2015); these factors included fatigue, depression, pain and distress (Berger, 1999; Ancoli‐Israel, 2009; Savard, Villa, Ivers, Simard, & Morin, 2009; Schutte‐Rodin, Broch, Buysee, Dorsey, & Sateia, 2008). Combinations of these conditions are well documented in the literature and have a strong association with treatment outcomes. A key condition for understanding the factors related to sleep disturbances in cancer patients is an understanding that the symptoms are concomitant; such joint occurrence is known as symptom clusters, which are defined as groups of interrelated symptoms (Rha & Lee, 2017) with a negative impact on patient survival. Some studies included (Bischel et al., 2016; Chen, Yub, & Yanga, 2008; Nishiura et al., 2015; Phillips et al., 2012; Romito et al., 2014; Saini et al., 2013) in the present review found several combined symptoms that significantly interfered with sleep quality. There is a correlation between poor sleep quality and depression, pain, fatigue, distress and anxiety (Nishiura et al., 2015; Romito et al., 2014). The symptoms of fatigue, pain, depression and distress were the most prevalent.

Pain and sleep are interrelated and have interconnected physiological mechanisms, such as the activation of the neurons of the ventrolateral preoptic nucleus, which project into the tuberomammillary nucleus and affect the perception of pain due to its interaction with the brainstem nuclei. As the hypothalamus is connected to the locus coeruleus, pain triggers a noradrenergic reaction that has an impact on the control of the sleep–wake cycle (Teixeira, Yeng, Rosi, & Yohinaga 2019). The authors reported pain intensity as a mediating factor between circadian rhythms and sleep quality (Ma, Chang, & Lin, 2014). In cancer patients, severe pain was significantly correlated with a higher overall sleep score (worse sleep quality) as assessed by the PSQI (*r* = .69, *p* < .01). In a study that evaluated risk factors for sleep disturbances among women with breast cancer, a higher pain score was associated with participant‐reported poor sleep (Overcash, Tan, Patel, & Noonan, 2018).

Cancer‐related fatigue (CRF), a common clinical symptom among oncology patients, often occurs at all stages in the disease trajectory. It is highly prevalent not only among patients in the treatment phase but also among survivors. It is estimated that 31% of fatigued oncology patients present insomnia as a frequent complaint. Despite its high prevalence, there is no universally accepted definition of CRF or a gold standard questionnaire to measure this concerning symptom. The National Comprehensive Cancer Network (NCCN) defines the symptom as a persistent and distressing subjective feeling of physical, emotional and/or cognitive tiredness or exhaustion related to cancer or its treatment that is not proportional to recent activity and that interferes with normal functioning (Berger et al., 2015). Research shows that interrupted sleep patterns coexist with fatigue and affect every aspect of the lives of cancer patients (Kwekkeboom et al., 2018). This conjunction between fatigue and interrupted sleep patterns further diminishes patient functioning and, as a consequence, has negative repercussions for quality of life. These symptoms have not been fully controlled in this population despite efforts to acquire greater knowledge about the underlying mechanisms (Horng‐Shiuann, Davis, & Natavio, 2012).

The pain, the tumour itself, the treatment modalities, the patient's environment, the patient's lifestyle, the hormonal changes, the cytokine changes and the psychiatric issues related to the diagnosis or treatment of cancer have a significant effect on the worsening of the sleep pattern and the likely occurrence simultaneous symptoms (Kaplow, 2005). These data are relevant since the improvement of one symptom contributes beneficially to the others, which confirms the theory of symptom clusters. It was observed that some characteristics, such as the presence of sleep difficulties and the lifestyle before the beginning of treatment, combined with depression and fatigue that appear during treatment were responsible for a small but significant proportion of the variation in overall sleep quality (Phillips et al., 2012). These pre‐existing altered sleep conditions tend to worsen with the onset of treatment. Modifiable risk factors among the predisposing factors for sleep pattern changes, such as smoking, sedentary lifestyle and social conditions, need to be better investigated in the cancer trajectory, although studies show positive correlations among these factors, especially those related to age (Beck et al., 2010; Bischel et al., 2016).

Controlling anxiety, a common symptom among oncology patients, may be an effective therapeutic approach to reducing sleep disturbance. A study of women with ovarian cancer observed that depression and anxiety were commonly identified with fatigue and sleep disturbances (Nho, Reul Kim, & Nam, 2017). These results indicate that sleep disturbance and psychoemotional symptoms should be carefully examined and managed simultaneously over the course of chemotherapy. It may be that during the treatment period, cancer patients have greater control over their level of anxiety, since interventions for this symptom in individuals without cancer have been shown to be related to lower sleep latency, with individuals with a more advanced age presenting better sleep quality (Gould, Beaudreau, O'Hara, & Edelstein, 2016).

A study compared healthy individuals to those with stage I and II cervical cancer undergoing chemotherapy and found altered sleep patterns in the latter group, especially among those receiving adjuvant treatment (Tian et al., 2015). Radiotherapy has an important effect on insomnia (Savard, Ivers, Savard, & Morin, 2015) and is significantly mediated by dyspnoea and night sweating, with greater effects in women with breast cancer.

In 2010, a meta‐synthesis (Enderlin et al., 2010) that summarized 10 cross‐sectional and 9 longitudinal studies on sleep disturbances in women with breast cancer undergoing chemotherapy did not show consistent results due to variations in sample size, type of population studied, lack of evaluation of the predictive factors and the limited number of time points evaluated throughout the studies. There were also inconsistencies in the evaluation of the alteration in sleep patterns during treatment, due to the diversity of the instruments used (Enderlin et al., 2010). These results are consistent with the findings of the present review, which found a lack of clinical trials and a wide variety of instruments used for sleep assessment. This lack of homogeneity makes it difficult to compare results and conduct care planning that can be incorporated into clinical practice (Enderlin et al., 2010; Mark et al., 2017).

The predisposing factors described in the evaluated studies (Beck et al., 2010; Bischel et al., 2016; Chen et al., 2008; Jim et al., 2011; Nishiura et al., 2015; Phillips et al., 2012; Romito et al., 2014; Saini et al., 2013; Tian et al., 2015) showed variation in important characteristics. Although ethnicity, living alone and educational level have been correlated with sleep disturbances (Beck et al., 2010; Bischel et al., 2016), another study that analysed cancer patients undergoing treatment did not show these associations (Innominato et al., 2012;). The decrease in physical capacity demonstrated in some studies (Romito et al., 2014; Tian et al., 2015) is a relevant and common problem, even though the nature and severity of the adverse consequences of treatment vary according to the type of cancer, the treatment regimen and the individual characteristics of the patients. Decreased physical capacity can affect a patient's ability to be fully functional at work, resulting in prolonged absences, suboptimal productivity and the total abandonment of work (Alfano, Kent, Padgett, Grimes, & de Moor, 2017). However, since most studies that have investigated physical functioning have significant biases that limit generalization, more research on this aspect is needed. The personal and social costs of physical, psychosocial and cognitive functioning problems that limit employment for individuals with cancer are modifiable impairments. There is a need to better identify these problems early and refer patients for cancer rehabilitation and related interventions so that adverse treatment complications are managed successfully, and physical capacity is preserved. It is necessary for the treatment of cancer to evolve to help patients remain optimally healthy, functional and employed (Alfano et al., 2017). In patients with breast cancer, poor sleep quality was associated with the worsening of health‐related quality of life (Sanford et al., 2013), a finding that was supported in the studies analysed in this review (Nishiura et al., 2015; Romito et al., 2014).

In the analysis of populations with other chronic diseases, the neurological disorder of restless legs syndrome has been identified as a relevant risk factor for altered sleep patterns (İn, Turgut, & Özdemir, 2016). In the present review, only one study investigated this variable; it deserves further study as the result showed a significant correlation with anxiety and worse quality of life (Saini et al., 2013).

A review study found that sleep should be evaluated in a multidimensional way; however, the gold standard for evaluating sleep‐related problems needs to be better defined (Loh, Burhenn, Hurria, Zachariah, & Mohile, 2016; Ritmala‐Castren, Lakanmaa, Virtanen, & Leino‐Kilpi, 2014). It is believed that a combination of objective and subjective measures is the best way to evaluate sleep patterns. Several questionnaires can be used for the rapid evaluation of insomnia symptoms and related problems (Schutte‐Rodin et al., 2008). The PSQI, which has good psychometric quality and internal consistency, is commonly used in research (Mollayeva et al., 2016). Among the 16 studies selected for the present review, 10 (62.5%) used the PSQI and three of these studies combined the PSQI with objective measures. As sleep is influenced by multidimensional factors, it can be inferred that studies that use both objective and subjective measures can demonstrate more consistent results.

Actigraphy, the second most commonly used instrument in the analysed studies, is the measure with the closest results to polysomnography, which is considered the best way of evaluating sleep quality. Due to the practicality of actigraphy for investigating sleep disturbance, its use is recommended for patients with different clinical conditions (Riemann et al., 2017). As there is good agreement among studies that report correlations between the results of actigraphy and polysomnography, studies that use actigraphy offer consistent data for its use. Symptoms such as fatigue, menopausal stage, advanced age and changes in inflammatory markers showed the best evidence when used in combination with subjective measures and objective actigraphy results (Beck et al., 2010; Liu et al., 2012; Rissling, Liu, Natarajan, He, & Ancoli‐Israel, 2011). Despite the improved readings of the sleep pattern when these two methods are combined, the agreement between the subjective and objective sleep efficiency in older people population was not similar, which suggest that even though actigraphy is recommended, it is not ideal for classifying sleep quality (Hughes et al., 2018).

This review found a large number of studies examining patients with breast cancer, as previously reported, which shows the concern with these breast cancer patients in light of the high worldwide prevalence of this disease (Brasil, 2014). However, in the meta‐synthesis conducted by Enderlin et al. (2010), which analysed sleep patterns in women with breast cancer, the results did not present good external validity. In that review, the population samples from the studies were small, were predominantly Caucasian, had a very large age range, did not include comparison groups or analyses that considered age differences and did not observe exclusion criteria or covariates, such as menopausal status, comorbidities or depression, which may have influenced the findings. In addition to the need to improve the quality of studies with women with breast cancer, it is essential to investigate differences in sleep patterns in populations of patients with other types of cancer; this is strongly recommended as a current research priority (Knobf et al., 2015).

Changes in the circadian cycle (Mansano‐Schlosser & Ceolim, 2017) were described in only one study; however, they represent an important risk factor that is exacerbated by chemotherapy and often associated with fatigue (Ortiz‐Tudela et al., 2014). These changes should be considered according to individual characteristics and may influence treatment outcomes and quality of life. Interventions related to sleep hygiene are recommended for sleep management according to the American Academy of Sleep Medicine (Schutte‐Rodin et al., 2008), but they need to be combined with other interventions, such as cognitive behavioural therapy and relaxation therapies (Howell et al., 2014). Such lines of treatment offer an important research opportunity for health professionals and are particularly applicable by the multiprofessional team, which facilitates the participation of other professionals in addressing the problem. The studies analysed in the present review showed increasing activity among medical professionals in this area of knowledge. Advancement in our understanding of circadian cycles requires examining the reasons that changes in sleep pattern are common in oncology patients undergoing chemotherapy and providing an interdisciplinary approach to their treatment.

The United States produced the greatest amount research on the subject of sleep disturbances in cancer patients undergoing chemotherapy, as is true in several other areas of research. Recent literature has reinforced the need to produce and implement research to foster knowledge in different countries and by different professionals as a matter public health policy throughout the world (Theobald et al., 2018). Because changes in sleep patterns are a global problem, there should be greater incentive to study this symptom in different populations of cancer patients as has been recommended by associations of different health and research professionals.

### 4.1 Implications for practice

The survey of the risk factors for sleep quality of patients undergoing chemotherapy indicates the need to implement interventions to control these symptoms. Such implementation is critical to improving the quality of life of affected individuals and influencing treatment efficacy and clinical outcomes. One of the limitations of the present review was that the therapeutic regimen of the treatments was not described in the studies, which could have provided a better understanding of the studied subject.

The nurses, as a reference professional engaged in care that prioritizes patient autonomy and centrality, are a determining element for the success of these goals, and they can develop and implement prevent and treatment's care.

### 4.2 Implications for research

Studies on the subject in populations that represent several types of cancer are needed so that their results can be widely applied. In addition to knowledge about the intensity of associations between factors and sleep disturbances among patients undergoing chemotherapy, prevention and health promotion interventions based on studies with methodological rigour are essential.

The gap in knowledge about the subject of this review indicates the need for a commitment among sleep quality researchers to reflect on the importance of conducting new studies that improve and maintain the health and well‐being of individuals, especially those with disadvantageous conditions (Ritmala‐Castren et al., 2014). It is necessary to identify common aetiological factors between sleep disturbances and other symptoms and to conduct longitudinal studies to examine their temporal relationships in cancer patients.

## 5 CONCLUSION

This review has made important contributions to understanding the factors that influence the sleep patterns of cancer patients undergoing chemotherapy. Sleep disruptions are multifactorial, and these factors are often present simultaneously, which makes their effective management difficult. However, due to this characteristic, when an intervention benefits one symptom, it improves others.

The most prevalent factors were those related to the disease and to treatment, such as fatigue, pain, depression, anxiety and distress. Predisposing factors related to lifestyle and demographic characteristics also have a significant correlation with sleep disturbances.

## AUTHOR CONTRIBUTIONS

Regina Claudia da Silva Souza contributed to research project elaboration, review of articles, search the databases, and manuscript writing. Ana Lucia Siqueira Costa Calache contributed to research project elaboration, manuscript writing, and study orientation. Maiara Rodrigues dos Santos, Izabel Alves das Chagas, and Cristina Silva Sousa contributed to review of articles and manuscript writing.

## ETHICAL APPROVAL

There is not Ethics Committee approval document because it is a literature review study.

## Supporting information

Table S1
